# Identification of the Key Enzymes in WL Gum Biosynthesis and Critical Composition in Viscosity Control

**DOI:** 10.3389/fbioe.2022.918687

**Published:** 2022-05-27

**Authors:** Hui Li, Zaimei Zhang, Jianlin Liu, Zhongrui Guo, Mengqi Chen, Benchao Li, Han Xue, Sixue Ji, Hang Li, Lijian Qin, Ling Zhu, Jiqian Wang, Hu Zhu

**Affiliations:** ^1^ State Key Laboratory of Heavy Oil Processing and Centre for Bioengineering and Biotechnology, College of Chemistry and Chemical Engineering, China University of Petroleum (East China), Qingdao, China; ^2^ Engineering Research Center of Industrial Biocatalysis, Fujian-Taiwan Science and Technology Cooperation Base of Biomedical Materials and Tissue Engineering, College of Chemistry and Materials Science, Fujian Normal University, Fuzhou, China; ^3^ College of Chemical Engineering and Materials Science, Quanzhou Normal University, Quanzhou, China

**Keywords:** Sphingomonas sp. WG, exopolysaccharide, sphingan WL gum, biosynthesis pathway, key enzymes, acetyl content, viscosity control

## Abstract

As an important microbial exopolysaccharide, the sphingan WL gum could be widely used in petroleum, food, and many other fields. However, its lower production is still limiting its wider application. Therefore, to gain insights into the bottlenecks of WL gum production by identifying the key enzymes in the WL gum biosynthesis pathway, more than 20 genes were over-expressed in *Sphingomonas* sp. WG and their effects on WL gum production and structure were investigated. Compared to the control strain, the WL gum production of *welB* over-expression strain was increased by 19.0 and 21.0% at 36 and 84 h, respectively. The WL gum production of both *atrB* and *atrD* over-expression strains reached 47 g/L, which was approximately 34.5% higher than that of the control strain at 36 h. Therefore, WelB, AtrB, and AtrD may be the key enzymes in WL production. Interestingly, the broth viscosity of most over-expression strains decreased, especially the *welJ* over-expression strain whose viscosity decreased by 99.3% at 84 h. Polysaccharides’ structural features were investigated to find the critical components in viscosity control. The uronic acid content and total sugar content was affected by only a few genes, therefore, uronic acid and total sugar content may be not the key composition. In comparison, the acetyl degrees were enhanced by over-expression of most genes, which meant that acetyl content may be the critical factor and negatively correlated with the apparent viscosity of WL gum. This work provides useful information on the understanding of the bottlenecks of WL gum biosynthesis and will be helpful for the construction of high WL gum-yielding strains and rheological property controlling in different industries.

## Introduction

WL gum produced by *Sphingomonas* sp. WG, belongs to one exopolysaccharide (EPS) family named sphingan. It showed high viscosity and tolerance to extreme environments such as high temperature, strong acid or alkali, and high salinity. Therefore, it can be widely used in petroleum, food, and other fields (Li et al., 2016a; Ji et al., 2020; Ji et al., 2022). However, the WL gum production levels and conversion efficiency of the carbon source of *Sphingomonas* sp. WG are much lower than xanthan gum and other commercially produced polysaccharides, which became a bottleneck of its application. Therefore, it was necessary to investigate the efficient production of WL gum.

Both the structures and biosynthesis pathway of sphingans such as gellan gum, welan gum, diutan gum, S-77 and WL gum showed high similarity (Schmid et al., 2014). The proposed biosynthesis pathway of those sphingans included three main steps: 1) synthesis of nucleotide-sugar precursor catalyzed by enzymes such as phosphoglucomutase (PGM), pyrophosphorylase (UGPase), UDP-Glc dehydrogenase (Li et al., 2019) and RmlA to RmlD. 2) tetrasaccharide repeating unit assembly involved specific glycosyltransferases. 3) polymerization and export through the general Wzx/Wzy-dependent pathway. Several genetically engineered strains were constructed to improve their production or properties. One commonly-used strategy was the over-expression of the genes in the pathway to enhance the carbon flux toward the final polymer products (Schmid et al., 2015). However, this strategy was proved to be successful only in some cases. For example, over-expression of the *pgm* gene that catalyzed the interversion of glucose-1-phosphate and glucose-6-phosphate in *Sphingomonas sanxanigenens* resulted in about 17 ± 0.3% increase in sphingan production (Huang et al., 2013). Similarly, the over-expression of *welK* that transferred the uronic acid (GlcA) to assemble the repeating unit in *Sphingomonas* sp. WG was also attributed to increased WL gum production (Li et al., 2021). In contrast, this strategy failed in some cases. Individual over-expression of *pgm* and *ugp* did not enhance gellan gum production significantly (Fialho et al., 2008). One important reason is that there was little information on the biosynthesis pathway’s bottlenecks which is crucial to the result of engineering or metabolic engineering.

The objective of this work was to gain insights into bottlenecks of WL gum production by identifying the key enzymes in the WL gum biosynthesis pathway. Therefore, more than 20 genes were cloned and over-expressed in *Sphingomonas* sp. WG. The fermentation of these over-expression strains was performed, and the effects of different gene over-expression on WL gum production were studied. Furthermore, the structures of EPS produced by different over-expression strains were also characterized to seek the critical composition for WL gum viscosity properties.

## Materials and Methods

### Strains, Plasmids, Culture Conditions, and Chemicals

The strains and plasmids used are listed in Supplementary Table S1. For genetic engineering, the WL gum-producing strain *Sphingomonas* sp. WG (CCTCC No. M2013161) was cultured in LB medium (10 g/L tryptone, 5 g/L yeast extract, 10 g/L NaCl). The *welK* over-expression strain was obtained in our previous work (Li et al., 2021). In the fermentation process, the strain and the related over-expression strains were activated in a seed medium containing Glc (10 g/L), yeast extract (1 g/L), and tryptone (5 g/L), KH_2_PO_4_ (2 g/L), and MgSO_4_ (0.1 g/L) at 28°C. After activation, the strain was transferred to 250 ml shaking flasks containing 50 ml fermentation medium (67 g/L Glc, 3.4 g/L yeast extract, 0.1 g/L MgSO_4_, 3 g/L K_2_HPO_4_, pH 7.0), and cultured at 32 °C, 150 rpm for 36, 60 and 84 h. To obtain the recombinant over-expression plasmids, the broad host plasmid pBBR1MCS-3 (Wuhan Miaoling Bioscience and Technology Co., Ltd.) (Kovach et al., 1995) was used, and *Escherichia coli* DH5α was chosen as the host strain. *E. coli* DH5α harboring the recombinant plasmid was cultivated in LB medium supplemented with tetracycline at 37 °C. Restriction enzymes and the DNA Ligation Kit were purchased from TaKaRa Biotechnology (Dalian, China). KOD FX DNA Polymerase was obtained from TOYOBO (shanghai) Biotech Co., Ltd. Primers were designed using Primer Premier software and synthesized by Shanghai Sangon Biotechnology (Shanghai, China). Tetracycline was obtained from Sigma Chemical Co. (St. Louis, MO, United States), and other reagents were purchased from Sinopharm Chemical Reagent Co., Ltd. (Shanghai, China).

### Construction of the Over-expression Strains

Firstly, the target gene fragments were obtained by PCR amplification using the *Sphingomonas* sp. WG genomic DNA as the template with the corresponding primers (Supplementary Table S2). The PCR products were gel-purified. Secondly, both the purified PCR products and the pBBR1MCS-3 plasmid were digested and ligated to obtain the recombinant plasmids that should be confirmed by colony PCR and DNA sequencing. Finally, the engineered strains with the recombinant plasmids were obtained by triparental conjugal mating in which the *E. coli* DH5α containing the recombinant plasmid, *E. coli* pRK 2013, *Sphingomonas* sp. WG was the donor, the helper, and recipient strains, respectively. Besides, the plasmid pBBR1MCS-3 was transformed into *Sphingomonas* sp. WG to obtain the control strain. The target strain was screened on LB medium supplemented with streptomycin (50 μg/ml) and tetracycline (10 μg/ml) and identified by colony PCR using the specific primers pMCS3-F/pMCS3-R for pMCS3 fragment of the plasmid pBBR1MCS-3 (Li et al., 2021).

### Measurement of Cell Growth, Broth Viscosity, and EPS Production of Different Over-expression Strains

To identify the possible key enzymes, the fermentation of over-expression strains and the control strain was performed in 50 ml fermentation medium in 250 ml shake flasks for 36, 60, and 84 h using the conditions described in our previous work (Li et al., 2018). Finally, the biomass OD_600_, the EPS production, and the viscosity of fermentation broth were measured as previously described (Cifuente et al., 2016; Li et al., 2018; Li et al., 2021). In particular, the EPS production was determined using the phenol-sulfuric acid colorimetric method with Glc as the standard (Zhou et al., 2017). The viscosity of the fermentation broth was determined using a Brookfield Viscometer DV-III (Brookfield Engineering Laboratories), and the rotor spindle LV3 was used at 1 rpm at 25°C.

### Compositional Analysis of EPS Produced by Different Over-expression Strains

The EPS samples produced by different strains cultured for 84 h were prepared by Lopes et al.(Lopes et al., 1994). The total sugar contents of different samples were detected by the phenol-sulfuric acid method (DuBois et al., 1956), and their GlcA contents were measured by the sulfuric acid-carbazole method (Bitter and Muir, 1962). The acetyl contents were determined as the previous report by Hestrin (Hestrin, 1949). The neutral monosaccharide composition analysis was performed using gas chromatography (GC) after hydrolyzing EPS to monosaccharides by trifluoroacetic acid. The obtained monosaccharides were further converted into acetylated aldononitriles (Chen et al., 2009). The GC analysis was conducted on an Agilent 7890A gas chromatograph (Agilent Technologies, Santa Clara, CA) and the detection conditions were as follows: the column: an HP-5 capillary column (30 m × 0.32 mm×0.25 μm film thickness); detector: flame ionization detector; the carrier gas: nitrogen; the injection volume: 1 μL; the injection temperature: 280°C; the detection temperature: 300°C; the temperature program: 150°C for 5 min, 150 to 250°C at a rate of 8°C per min, and 250°C for 5 min; the split ratio at 1:10 (v/v). Monosaccharides such as Glc, mannose (Man), galactose (Gal), and rhamnose (Rha) were used as the standards and they were also converted into their acetylated aldononitriles to calculate the percentage of each monosaccharide. Inositol was used as the internal reference.

## Results and Discussion

### Construction of Over-expression Strains

**FIGURE 1:**
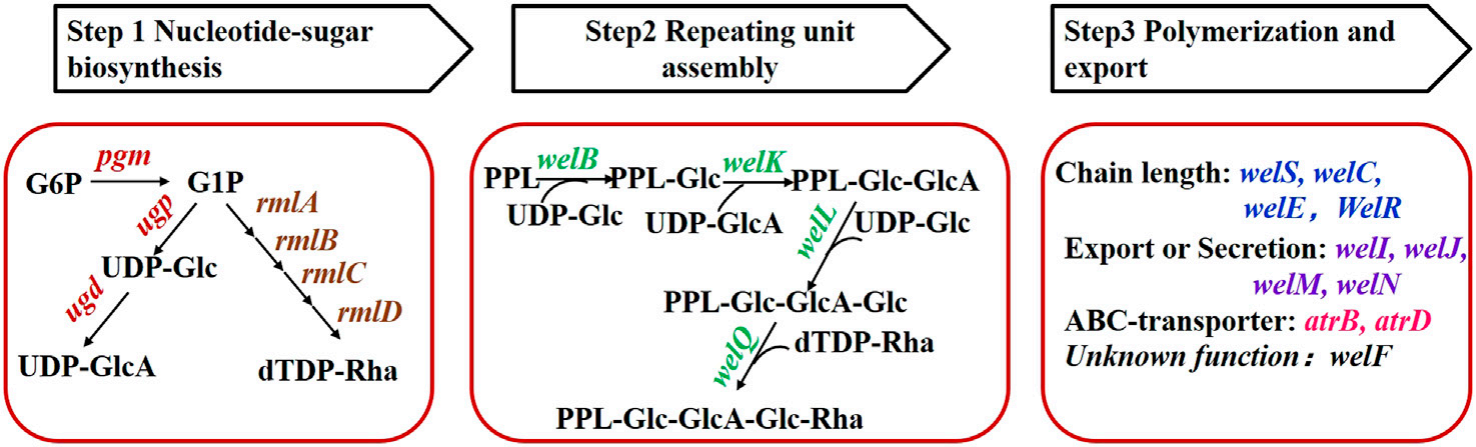
Grouping of genes involved in WL gum biosynthesis. G6P: glucose-6-phosphate, G1P: glucose-1-phosphate, PPL: pyrophosphorylpolyprenol.

According to the proposed biosynthesis pathway (Li et al., 2016b), more than 20 genes were involved in the biosynthesis of WL gum and were divided into seven groups (Figure 1) on the basis of their functions. Those genes were cloned into the pBBR1MCS-3 vector using the corresponding primers to obtain the over-expression plasmids. The electrophoresis results (Supplementary Figure S1) showed that the sizes of fragments obtained from PCR verification using recombinant plasmids as templates were consistent with the expectations. Furthermore, sequencing results also demonstrated that all target genes had been cloned into the pBBR1MCS-3 vector. Finally, the over-expression strains harboring the recombinant plasmid were constructed via the triparental conjugation mating method (Supplementary Figure S2). Fermentation of the different over-expression strains was carried out to compare their influence on cell growth and WL gum production.

### The Effects of Genes in Nucleotide-sugar Biosynthesis on Biomass and WL Gum Production

**FIGURE 2:**
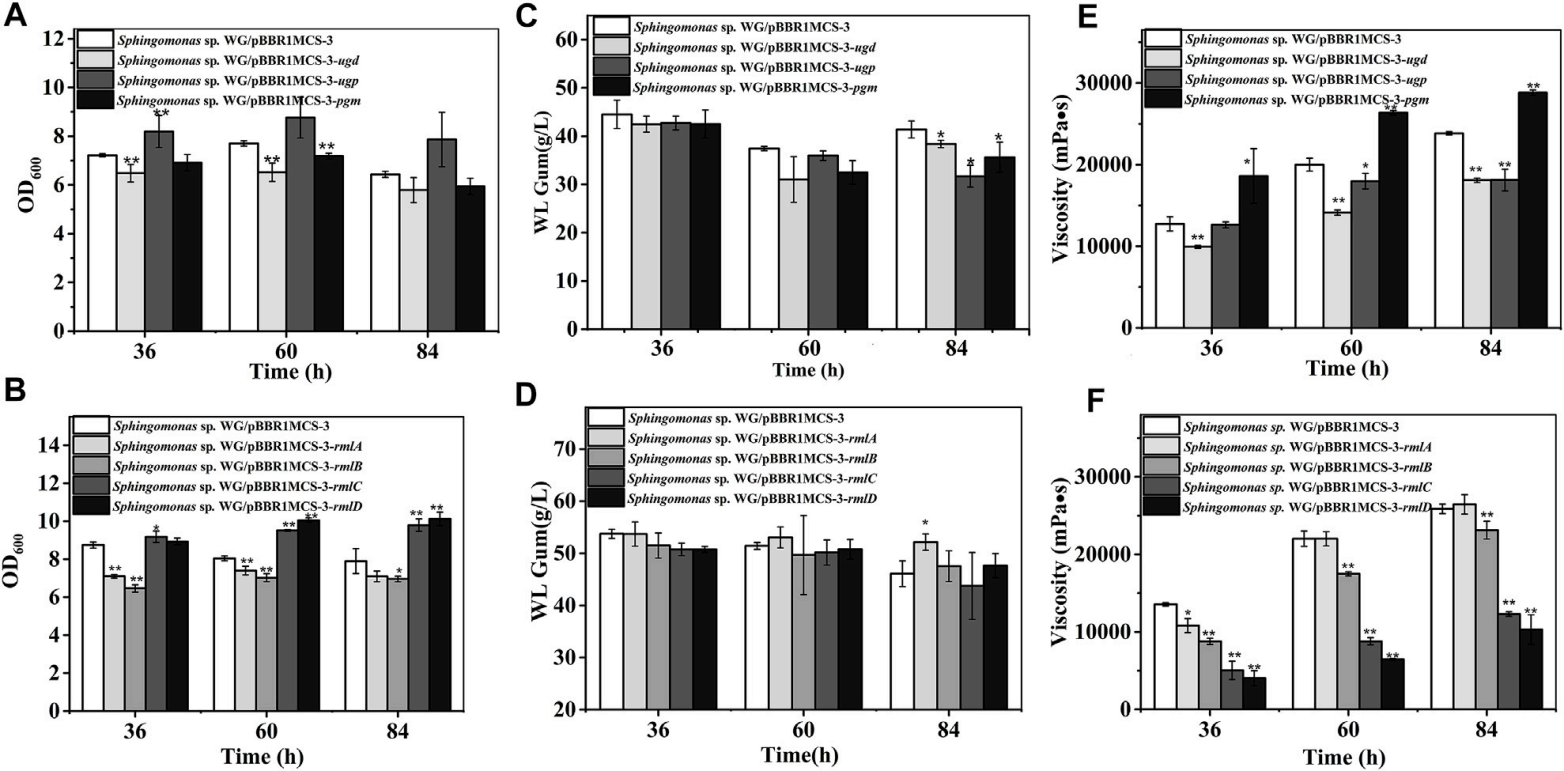
Fermentation results of over-expression strains containing genes involved in nucleotide-sugar biosynthesis. **(A)** Comparison of biomass of *Sphingomonas* sp. WG/pBBR1MCS-3-*pgm*, *Sphingomonas* sp. WG/pBBR1MCS-3-*ugp,* and *Sphingomonas* sp. WG/pBBR1MCS-3-*ugd*. **(B)** Comparison of biomass of *Sphingomonas* sp. WG/pBBR1MCS-3-*rmlA*, *Sphingomonas* sp. WG/pBBR1MCS-3-*rmlB, Sphingomonas* sp. WG/pBBR1MCS-3-*rmlC*, and *Sphingomonas* sp. WG/pBBR1MCS-3-*rmlD*. **(C)** Comparison of WL gum production of *Sphingomonas* sp. WG/pBBR1MCS-3-*pgm*, *Sphingomonas* sp. WG/pBBR1MCS-3-*ugp,* and *Sphingomonas* sp. WG/pBBR1MCS-3-*ugd*. **(D)** Comparison of WL gum production of *Sphingomonas* sp. WG/pBBR1MCS-3-*rmlA*, *Sphingomonas* sp. WG/pBBR1MCS-3-*rmlB, Sphingomonas* sp. WG/pBBR1MCS-3-*rmlC*, and *Sphingomonas* sp. WG/pBBR1MCS-3-*rmlD*. **(E)** Comparison of fermentation broth viscosity of *Sphingomonas* sp. WG/pBBR1MCS-3-*pgm, Sphingomonas* sp. WG/pBBR1MCS-3*-ugp,* and *Sphingomonas* sp. WG/pBBR1MCS-3*-ugd*. **(F)** Comparison of fermentation broth viscosity of *Sphingomonas* sp. WG/pBBR1MCS-3-*rmlA*, *Sphingomonas* sp. WG/pBBR1MCS-3-*rmlB, Sphingomonas* sp. WG/pBBR1MCS-3-*rmlC*, and *Sphingomonas* sp. WG/pBBR1MCS-3-*rmlD*. The fermentation was preformed in 250 ml shaking flasks containing 50 ml fermentation medium (67 g/L Glc, 3.4 g/L yeast extract, 0.1 g/L MgSO_4_, 3 g/L K_2_HPO_4_, pH 7.0), and cultured at 32 °C, 150 rpm for 36, 60 and 84 h. The strain *Sphingomonas* sp. WG/pBBR1MCS-3 was used as the control.

The first step in WL gum biosynthesis is the generation of activated nucleotide sugar precursors. The genes related to sugar nucleotide biosynthesis are the targets in many types of research to increase the carbon flux toward the final EPS product (Schmid et al., 2015). The effects of those genes such as *pgm*, *ugp,* and *ugd* involved in the biosynthesis of UDP-Glc and UDP-GlcA and *rmlA* to *rmlD* involved in the formation of dTDP-Rha were investigated. As shown in Figure 2, almost all of these genes influenced the cell growth of *Sphingomonas* sp. WG. In particular, the over-expression of *ugp, rmlC,* and *rmlD* enhanced the biomass. Differently, the over-expression of *ugd*, *pgm, rmlA,* and *rmlB* led to the lower biomass.

Besides, the EPS-producing capacity of different recombinant strains was investigated through the fermentation of WL gum. Normally, the key enzymes may be enzymes that catalyzed the initial step in the special pathway. For example, the squalene epoxidase catalyzing the first oxygenation step in the triterpenoid and phytosterol biosynthetic pathway is one of the key enzymes in this pathway (Gao et al., 2016). Tyrosinase catalyzed both the first and key step in melanin production (Wachamo et al., 2021). PGM catalyzed the branch point reaction-the interconversion of glucose-1-phosphate from glucose-6-phosphate in carbohydrate metabolism. Therefore, it might be the key enzyme in sphingan production. However, the over-expression of *pgm* in *Sphingomonas elodea* ATCC 31461 showed no positive effect on gellan production (Fialho et al., 2008). Similarly, the over-expression of *ugp* in *Sphingomonas elodea* ATCC 31461 did not enhance gellan production (Fialho et al., 2008), although UGPase is vital for forming a UDP-Glc precursor. UGP1 is the key enzyme in pullan biosynthesis (Yang et al., 2020). As shown in Figure 2, the over-expression of *pgm*, *ugp,* and *ugd* did not increase WL gum production. In contrast, they showed a negative effect when cultured for 84 h. Similarly, the WL gum-producing capacity of the over-expression strains of genes *rmlB*, *rmlC,* and *rmlD* did not change significantly except for the *rmlA* over-expression strain, whose WL gum production was 13.0% higher than that of the control strain when cultured for 84 h. Those results indicated that the biosynthesis of nucleotide sugar precursors was not the key step in WL gum biosynthesis.

Viscosity is another important property of the EPS. Therefore, the viscosity of the fermentation broth was also detected (Figure 2). It was obvious that most over-expression strains showed dramatically decreased viscosity. The exception was the *Sphingomonas* sp. WG/pBBR1MCS-3-*pgm* whose fermentation broth viscosity was 1.4-fold, 1.3-fold, and 1.2-fold compared with control at 36, 60, and 84 h, respectively.

### The Effects of Genes in the Repeating Unit Assembly on Biomass and WL Gum Production

**FIGURE 3:**
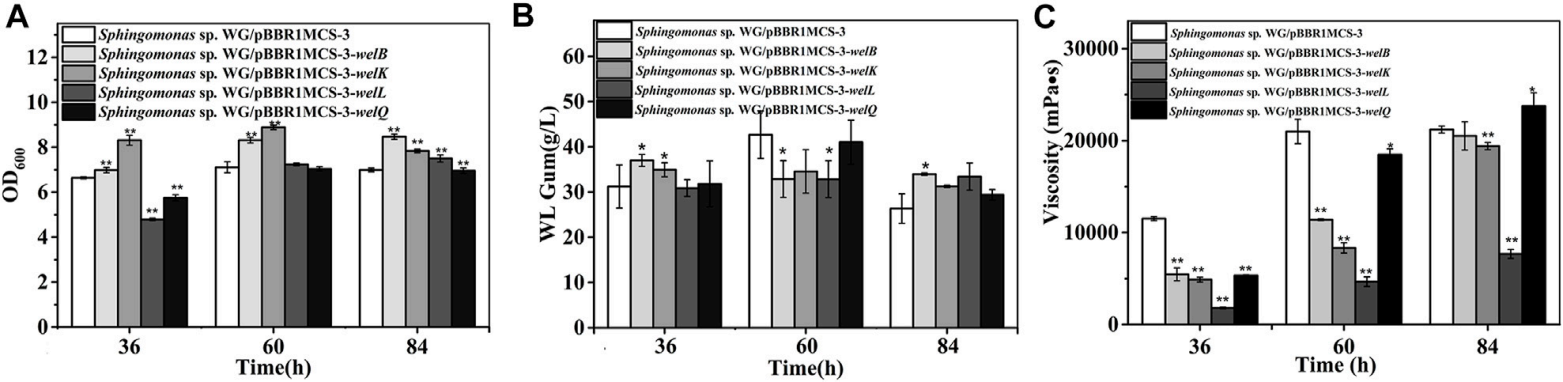
Fermentation results of over-expression strain *Sphingomonas* sp. WG/pBBR1MCS-3-*welB*, *Sphingomonas* sp. WG/pBBR1MCS-3-*welK*, *Sphingomonas* sp. WG/pBBR1MCS-3-*welL*, *Sphingomonas* sp. WG/pBBR1MCS-3-*welQ* that contained genes involved in repeating unit assembly process. **(A)** Comparison of biomass of different over-expression strains. **(B)** Comparison of WL gum production of different over-expression strains. **(C)** Comparison of fermentation broth viscosity of different over-expression strains. The fermentation was preformed in 250 ml shaking flasks containing 50 ml fermentation medium (67 g/L Glc, 3.4 g/L yeast extract, 0.1 g/L MgSO_4_, 3 g/L K_2_HPO_4_, pH 7.0), and cultured at 32 °C, 150 rpm for 36, 60 and 84 h. The strain *Sphingomonas* sp. WG/pBBR1MCS-3 was used as the control.

The tetrasaccharide repeating unit assembling is the second step in WL gum biosynthesis, and the genes in this process, including *welB*, *welK*, *welL,* and *welQ* were also very important. The over-expression of *welB* and *welK* encoding the priming and second glycosyltransferases that transfer Glc and GlcA showed positive effects on cell growth (Figure 3). The biomass of the third and fourth transferase encoding genes *welL* and *welQ* over-expression strains was significantly lower than the control group at 36 h. However, when cultured for 84 h, their biomass was higher. This result indicated that the over-expression of *welL* and *welQ* also showed a positive effect on biomass finally, although the strains’ growth rate was slower at the early stage.

The yield of *Sphingomonas* sp. WG/pBBR1MCS-3-*welB* was 19.0%, 21.0% higher than that of the control group at 36 h, 84 h. Considering that *welB* encodes the priming transferase, it is probably that the first step in the repeating unit assembly is one of the limiting steps in WL gum production. Similar to our previous work, the over-expression of *welK* enhanced the WL gum production at 36 h (Li et al., 2021). When cultured for 84 h, WL gum production of the over-expression strains of *welK*, *welL* and *welQ* showed no obvious changes.

As the fermentation proceeded, the fermentation broth viscosity of different strains was increased. Still, the broth viscosity of all over-expression strains, especially the *welL*-over-expression strain, was much lower than the control group at 36 and 60 h. Interestingly, the viscosity of the *welQ* over-expression strain was increased by 10.7%, while that of *welL* over-expression was much lower (approximately 63.8% of the control strain) at 84 h. WelL and WelQ transfer the third Glc and the fourth Rha to the repeating unit. It was reasonable that the over-expression of these genes led to composition changes of the EPS products thereby resulting in the increased or decreased viscosity.

### The Effects of Genes in Polymerization and Exportation Process on Biomass and WL Gum Production

**FIGURE 4:**
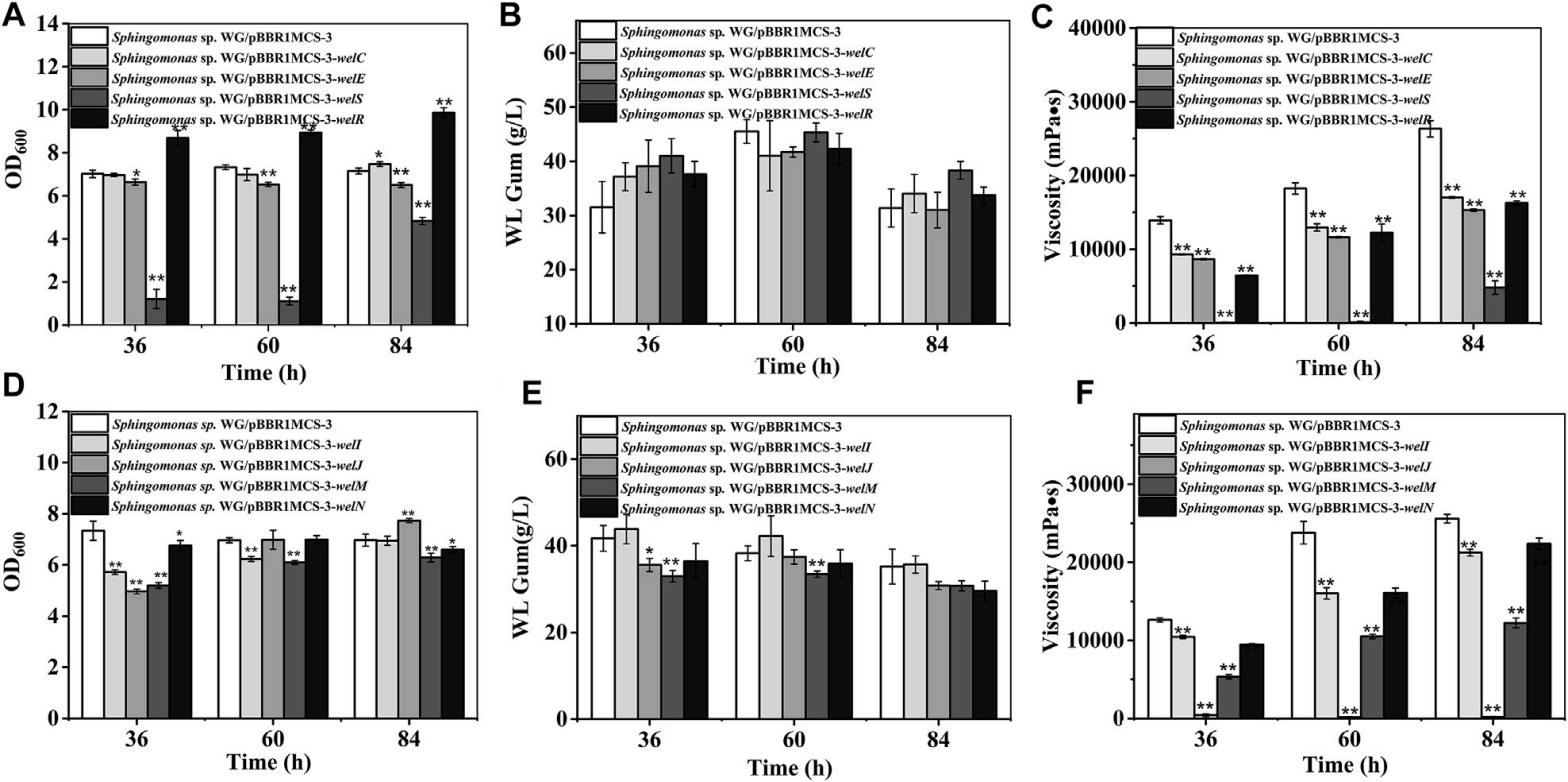
Fermentation results of over-expression strains containing genes involved in polymerization and export process. **(A)** Comparison of biomass of *Sphingomonas* sp. WG/pBBR1MCS-3-*welC*, *Sphingomonas* sp. WG/pBBR1MCS-3-*welE, Sphingomonas* sp. WG/pBBR1MCS-3-*welS*, and *Sphingomonas* sp. WG/pBBR1MCS-3-*welR*. **(B)** Comparison of WL gum production of *Sphingomonas* sp. WG/pBBR1MCS-3-*welC*, *Sphingomonas* sp. WG/pBBR1MCS-3-*welE, Sphingomonas* sp. WG/pBBR1MCS-3-*welS*, and *Sphingomonas* sp. WG/pBBR1MCS-3-*welR*. **(C)** Comparison of fermentation broth viscosity of *Sphingomonas* sp. WG/pBBR1MCS-3-*welC*, *Sphingomonas* sp. WG/pBBR1MCS-3-*welE, Sphingomonas* sp. WG/pBBR1MCS-3-*welS*, and *Sphingomonas* sp. WG/pBBR1MCS-3-*welR*. **(D)** Comparison of biomass of *Sphingomonas* sp. WG/pBBR1MCS-3-*welI*, *Sphingomonas* sp. WG/pBBR1MCS-3-*welJ, Sphingomonas* sp. WG/pBBR1MCS-3-*welM*, and *Sphingomonas* sp. WG/pBBR1MCS-3-*welN*. **(E)** Comparison of WL gum production of *Sphingomonas* sp. WG/pBBR1MCS-3-*welI*, *Sphingomonas* sp. WG/pBBR1MCS-3-*welJ, Sphingomonas* sp. WG/pBBR1MCS-3-*welM*, and *Sphingomonas* sp. WG/pBBR1MCS-3-*welN*. **(F)** Comparison of fermentation broth viscosity of *Sphingomonas* sp. WG/pBBR1MCS-3-*welI*, *Sphingomonas* sp. WG/pBBR1MCS-3-*welJ, Sphingomonas* sp. WG/pBBR1MCS-3-*welM*, and *Sphingomonas* sp. WG/pBBR1MCS-3-*welN*. The fermentation was preformed in 250 ml shaking flasks containing 50 ml fermentation medium (67 g/L Glc, 3.4 g/L yeast extract, 0.1 g/L MgSO_4_, 3 g/L K_2_HPO_4_, pH 7.0), and cultured at 32°C, 150 rpm for 36, 60 and 84 h. The strain *Sphingomonas* sp. WG/pBBR1MCS-3 was used as the control.

WL gum biosynthesis is a typical Wzx/Wzy dependent pathway. WelS is the homolog of the translocase Wzx; therefore, it might be related to the transportation of repeating units. The polymerization of the repeating units was presumed to be catalyzed by the polymerases WelG. The chain length of WL gum may be regulated by WelC and WelE. WelC contained a central periplasmic segment and two flanking transmembrane regions in the N terminal region. According to the SMART analysis, WelC contained a Wzz domain that was supposed to control the chain length of polysaccharides by affecting the polymerase assembly (Tocilj et al., 2008; Wiseman et al., 2021). WelE was homologous to the kinase domain of tyrosine autokinases, and it may have ATPase activity. Both WelC and WelE showed high identity to GelC and GelE (higher than 75%) in gellan gum biosynthesis. The deletion of *gelC* and *gelE* led to the abolishment of gellan gum and changed the composition and viscosity properties of the resulting products (Moreira et al., 2004). GelC and GelE regulated the gellan gum chain length concurrently (Moreira et al., 2004). WelR has been proved to be the specific polysaccharide lyase (Chang et al., 2021) and can degrade WL gum to small molecules. Considering their roles in polymerization process, *welG*, *welS*, *welC, welE,* and *welR* were divided into one group and their effects were investigated (Figure 4). The phenomenon that we could not obtain the over-expression strain of *welG* suggested that the higher expression of the polysaccharide polymerase might have a lethal effect. Similarly, the cell growth of the *welS* over-expression strain was much lower than that of the control group. The over-expression of *welS* may result in the accumulation of lipid-linked intermediates in the cytoplasmic membrane, which will harm cell growth because the carrier could not be released for other essential cellular functions (Pollock et al., 1998; Harding et al., 2004). In contrast, the over-expression of *welR* achieved the best biomass among these genes, which is attributed to the more sugars obtained from hydrolysis of the WL gum catalyzed by WelR (Chang et al., 2021). The over-expression of these genes showed no significant changes in WL gum production. One interesting phenomenon was that the WL gum production of *welS* over-expression strain was not significantly decreased compared with that of the control group, although *welS* inhibited the cell growth. Beyond our expectations, the over-expression of genes in this group that might affect the molecular weight did not enhance the broth viscosity.

In contrast, the viscosity decreased. WelS was proposed to translocate the repeating unit; therefore, perphaps it did not directly influence the chain length. The over-expression of the potential genes *dpsC* and *dpsE* also failed to increase the viscosity of diutan gum (Coleman et al., 2008). Differently, the over-expression of the polymerase Wzy resulted in a longer O-antigen chain length (Daniels et al., 1998). The over-expression of the PCP protein GumC led to a higher molecular weight of xanthan gum with higher intrinsic viscosity only at the proper inducer concentration (Galván et al., 2012). Therefore, we supposed *welC* or *welE* may affect the molecular weight only when they were at the proper level or simultaneously over-expressed. Besides the molecular weight, many factors, such as the sugar composition, and the acetyl degree might also affect the viscosity.

Potential genes involved in exporting WL gum, such as *welI*, *welJ*, *welM,* and *welN* are divided into another group (Figure 4). WelI, WelM, and WelJ might play roles in the polysaccharide attachment to the cell. The homolog of WelI, GelI in gellan gum biosynthesis might function in protein folding and export due to its weak homologous to peptidyl-prolyl cis-trans isomerase. A deletion of *gelI* led to the lowered gellan gum production but did not affect gellan gum composition, such as sugar composition or the acetylation degree (Harding et al., 2004). Another protein, WelN, showed high identity to GelN and SpsN. Those proteins have been predicted to participate in protein export because they showed high identity to EspI family protein (Yoshida et al., 2003; Fialho et al., 2008). The gellan gum yield and viscosity of GelM-GelN mutant were 78% and 30% of that of the wild-type strain (Harding et al., 2004). The predicted protein WelJ showed similarity to the AAA-superfamily of ATPases (CD0009) and was presumed to be another protein involved with WL gum secretion and transport. They showed complicated effects on cell growth. The inhibitory influence of *welI* and *welJ* on cell growth mainly at the early stage (36 h). *welM* showed negative effects throughout the culture process. Among the four different recombinant strains in this group, the WL gum production of *welI* and *welN* over-expression strains showed no significant increase; *welJ* and *welM* even decreased at 36 and 60 h. The over-expression of *gelN* in *S. elodea* and *Sphingobium chungbukense* DJ77 enhanced the production of related EPS (Lee et al., 2017; Lee et al., 2018). The different results might be due to the gene expression levels related to the used plasmids or the special biosynthesis mechanism of WL gum. Similar to other over-expression strains, the viscosity of the fermentation broth was decreased.

**FIGURE 5:**
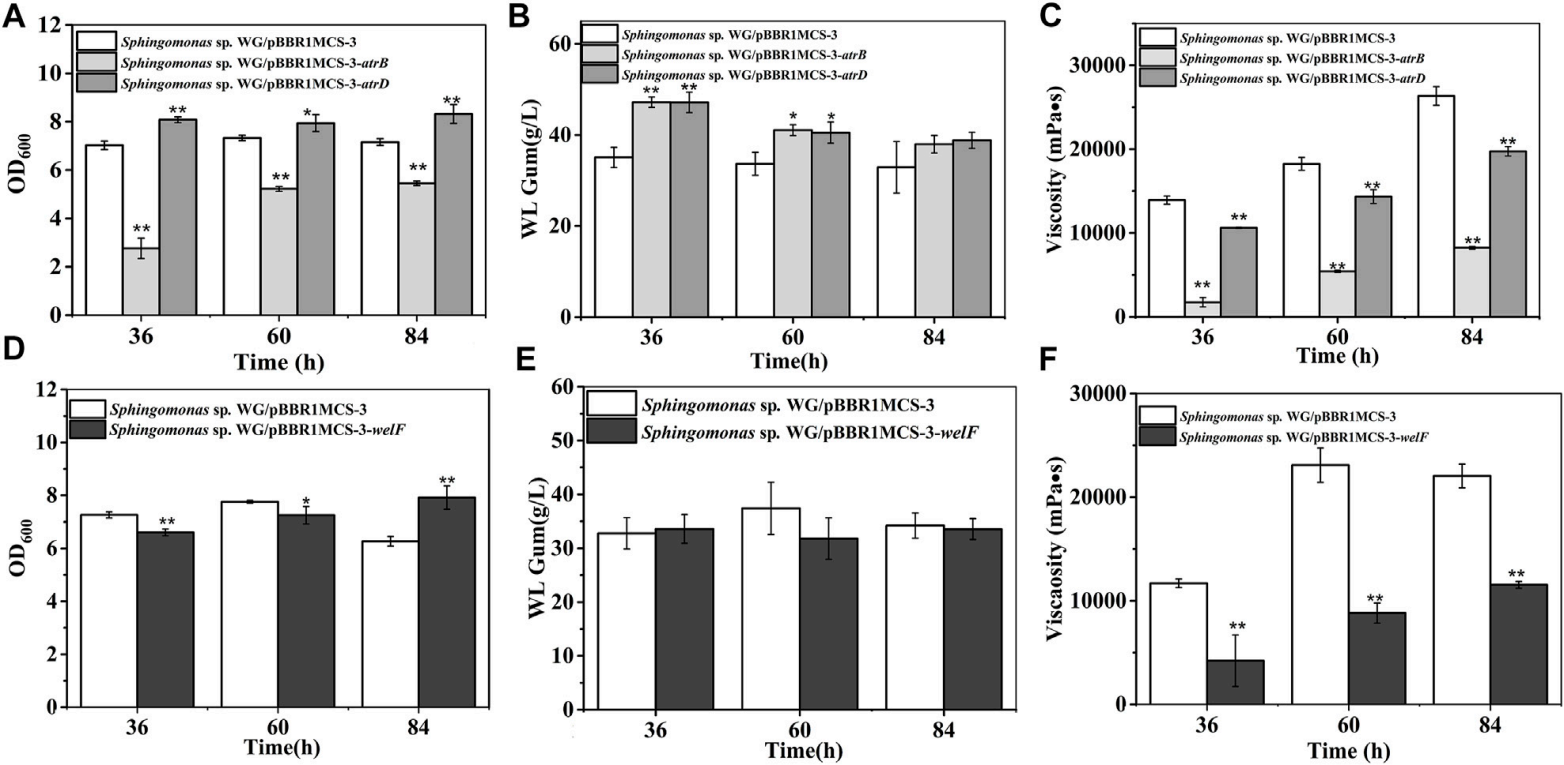
Fermentation results of over-expression strains containing *atrB*, *atrD,* or *welF*. **(A)** Comparison of biomass of *Sphingomonas* sp. WG/pBBR1MCS-3-*atrB* and *Sphingomonas* sp. WG/pBBR1MCS-3-*atrD*. **(B)** Comparison of WL gum production of *Sphingomonas* sp. WG/pBBR1MCS-3-*atrB* and *Sphingomonas* sp. WG/pBBR1MCS-3-*atrD*. **(C)** Comparison of fermentation broth viscosity of *Sphingomonas* sp. WG/pBBR1MCS-3-*atrB* and *Sphingomonas* sp. WG/pBBR1MCS-3-*atrD*. **(D)** Biomass of *Sphingomonas* sp. WG/pBBR1MCS-3-*welF*. **(E)** WL gum production of *Sphingomonas* sp. WG/pBBR1MCS-3-*welF*. **(F)** Fermentation broth viscosity of *Sphingomonas* sp. WG/pBBR1MCS-3-*welF*. The fermentation was preformed in 250 ml shaking flasks containing 50 ml fermentation medium (67 g/L Glc, 3.4 g/L yeast extract, 0.1 g/L MgSO_4_, 3 g/L K_2_HPO_4_, pH 7.0), and cultured at 32°C, 150 rpm for 36, 60 and 84 h. The strain *Sphingomonas* sp. WG/pBBR1MCS-3 was used as the control.

AtrB and AtrD showed identity to permease/ATPase/HylB and HlyD family protein in the type-I secretion system, respectively. They were supposed to involve in the transport of polysaccharides. They showed different effects on cell growth: AtrB inhibited cell growth while AtrD enhanced growth (Figure 5). The most interesting results were that the *atrB*, *atrD* over-expression strains produced more WL gum. The WL gum production of both *atrB* and *atrD* over-expression strain reached 47 g/L, which was increased by 34.5% at 36 h, respectively. These results meant that AtrB and AtrD were the key enzymes in WL gum biosynthesis. In some Gram-negative bacteria, the type-I secretion system permease/ATPase protein could export proteins (properly proteases) to the extracellular medium from internal and external membranes by utilizing the energy generated by ATP hydrolysis (Perez-Acosta et al., 2018). HlyD family protein is one kind of membrane fusion protein, and HlyD is a periplasmic adaptor protein that forms a continuous channel by docking HlyB and TolC (Lee et al., 2012). The type-I secretion system might participate in many processes such as uptake of nutrients, cell detoxification, and export of proteins, drugs, and capsular polysaccharides in many Gram-negative bacteria (Willis and Whitfield, 2013). AtrB and AtrD may involve the export of polysaccharides or related proteins or the intake of nutrients. Therefore, they enhanced the WL gum production in the liquid fermentation broth. Both strains showed lower viscosity than the control strain. The fermentation broth viscosity of *atrD* over-expression strain was much higher than that of *atrB* over-expression strain. Considering their different effects on cell growth and viscosity, they may have different roles, which will be investigated in our next experiment.

WelF might be an essential protein for WL gum production because it showed similarity (about 61%) to GelF in gellan gum biosynthesis, whose mutation was lethal. The over-expression effect of *welF* on WL gum production was investigated (Figure 5). The results showed that the biomass of the over-expression strain was lower than the control strain during the first 60 h, but the final biomass of the over-expression strain was increased than control as the control strain was in the decline phase at 84 h. The over-expression of *welF* did not show significant effects on WL gum production. Similar to other strains, the fermentation broth viscosity of the *welF* over-expression strain was also decreased significantly.

### The Composition Analysis of EPS Produced by Different Recombinant Strains

The rheological properties of WL gum were important due to their roles in its application in EOR, ink, and the food industry. The fermentation broth viscosity of most over-expression strains (except for *Sphinomanos* sp. WG/pBBR1MCS-3-*pgm*) was decreased. However, their WL gum concentration was not reduced significantly, which might be ascribed to the variation in the molecular properties such as the acetyl content, monosaccharide composition, and molecular weight (Schmid et al., 2015). Therefore, the structures of those EPS were investigated to find the key factors in viscosity control. The products of the strain *Sphingomonas* sp. WG/pBBR1MCS-3 was used as the control and named WL-Blank; the over-expression strain products were named as WL-the gene name, for example, the WL gum synthesized by *Sphingomonas* sp. WG/pBBR1MCS-3-*pgm* was named as WL-Pgm.

**FIGURE 6:**
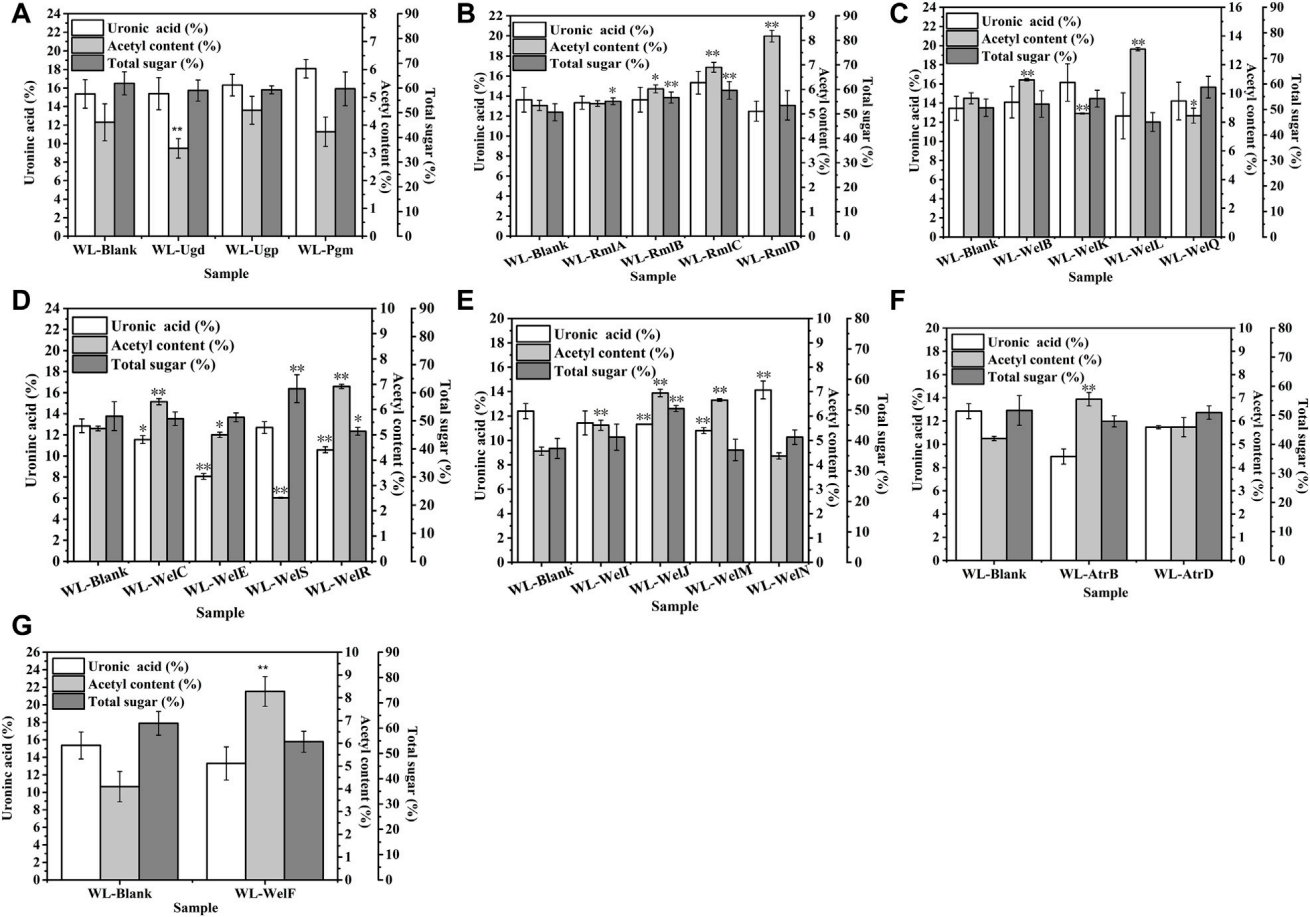
The effects of gene over-expression on the chemical composition of EPS. **(A)** The effects of genes in nucleotide-sugar UDP-glucose and UDP- glucuronic acid biosynthesis. **(B)** The effects of genes in nucleotide-sugar TDP-Rhamnose biosynthesis. **(C)** The effects of genes in repeating unit assembly. **(D)** The effects of genes in the polymerization process. **(E)** The effects of genes in exporting process. **(F)** The effects of ABC transporter protein AtrB and AtrD. **(G)** The effects of *welF* on the chemical composition of EPS.

As shown in Figure 6, similar to our previous work (Li et al., 2021)**,** the biosynthesis of WL gum was regulated in a complicated way (Li et al., 2022). Therefore, the over-expression of a single gene might cause a change in the carbon metabolic flux and thereby affect the EPS composition. Special genes changed the GlcA content: *welK*, *welN,* and *welJ* increased the GlcA content while four genes *ugd*, *welM*, *welE,* and *atrB* decreased the GlcA content. It was proposed that the intramolecular associations of many sphingans mainly consisted of hydrogen bonds that formed by the OH-4 of the D-glucopyranosyl residue and the adjacent hemiacetal oxygen atom of the l-rhamnopyranosyl residue and van der Waals forces between the methyl group of the l-rhamnopyranosyl residue and the adjacent hemiacetal oxygen atom of the D-glucopyranosyl residue (Tako, 2015; Xu et al., 2019). Therefore, the GlcA content and Rha content changes may influence the intramolecular associations and then affect their rheological properties. However, different from the fact that most recombinant strains’ viscosity was decreased, the over-expression of most genes did not change the GlcA content significantly. It indicated that the GlcA content might not be the key factor for viscosity.

Similarly, the total sugar content of most over-expression strains was not changed except *welJ, welS, rmlA-rmlD,* and *welR. welJ, welS, rmlA-rmlD* enhanced the total sugar content of WL gum to different degrees. For example, the total sugar content of WL-WelJ and WL-WelS was significantly increased by 34.9 and 19.9%, respectively. The total sugar content of WL-WelR was reduced by 10.5%. The results also suggested that the total sugar content was not the key factor for viscosity.

One interesting phenomenon was that the acetyl content of most over-expression strains increased significantly. For example, the acetyl content of WL-WelI, WL-WelJ, and WL-WelN was increased by 23.2, 52.2, and 45.8%, respectively. Harding et al. (Harding et al., 2004) found that the deletion of *gelI*, *gelM,* and *gelN* from the genome of *S. elodea* ATCC 31461 resulted in the decreased acetyl content. These genes affected the acetyl modification process, and the acetyl content played important roles in viscosity control. Two different insights on the effect of the acetyl group have been reported. Acetyl substituent has been proved to greatly influence the gelling process of another sphingan gum, gellan gum. The higher acetyl gellan gum could form a soft, elastic thermoreversible gel, while the lower or even deacyl gellan gum formed a harder, brittle one (Jay et al., 1998). It was inferred that the acetyl groups locate on the periphery of the double-helix formed by native gellan gum and thus prevent aggregation (Morris et al., 1996). In the other respect, the acetyl group might reduce the charge density and increase the flexibility of molecular bundles. Therefore, the interhelical association was enhanced, and the viscoelastic properties of gellan gum increased (Noda et al., 2008). Similarly, the increase in acetyl degree of alginates from 0 to 0.105 led to the rapid increase of the intrinsic viscosity (Skjåk-Bræk et al., 1989). However, the genetic engineering of xanthan biosynthesis demonstrated that more acetyl groups reduced the viscosity of the resulting EPS (Hassler and Doherty, 1990). Interestingly, our foundation supported the later insight that the acetyl content might negatively correlate with the apparent viscosity of WL gum; that is, in most cases, when the acetyl content was increased, the viscosity of WL gum was decreased.

Polysaccharides’ structural features and conformations might influence their properties. Similar to previous work (Thorne et al., 2000; Sun et al., 2007; Li et al., 2021), the over-expression of a single gene affected the overall sugar profile of WL gum (Supplementary Figure S3). Many genes such as *ugd*, *pgm*, *welJ*, *welC*, *welS*, *welE*, *atrB,* and *atrD* enhanced the Rha content, while a few genes such as *rmlA-D*, *welN,* etc. reduced the Rha content. Similarly, the Glc, Man, and Gal contents of many WL gum samples were changed. The relationship between the neutral monosaccharide composition and the viscosity of WL gum was not observed in this work. Our next work will investigate the structure-rheological properties relationship using these samples and their derivatives. Of course, molecular weight was another important factor that influenced the viscosity of many polysaccharides. The viscous flow of the WL gum was caused by the shifts of the molecular chain’s center of gravity along the flow direction and the slides between molecular chains through segment movement. As the molecular weight increased, the molecular chain contained more segments, suggesting that more synergistic segment shifts and slides were needed to accomplish the center of gravity shift. Presumably, the viscosity of WL gum increased with the increased molecular weight.

## Conclusion

More than 20 genes were over-expressed to gain insights into bottlenecks of WL gum production. Over-expression of *welB, atrB,* and *atrD* enhanced the yield of sphingan WL gum significantly, suggesting that there may be three key steps in the WL gum synthesis process. These genes might be the genetic engineering targets in constructing high WL gum-yielding strains. Besides, acetyl degrees showed a negative effect on WL gum viscosity. *pgm* and *welQ* increased the viscosity while most genes reduced the viscosity to different degrees, which will be helpful for the rheological property controlling in different industries.

## Data Availability

The datasets presented in this study can be found in online repositories. The names of the repository/repositories and accession number(s) can be found below: https://www.ncbi.nlm.nih.gov/genbank/, LNOS00000000.

## Abbreviations

EPS: exopolysaccharide, PGM: phosphoglucomutase, UGPase: pyrophosphorylase, RmlA: glucose-1-phosphate thymidilyltransferase; RmlB: dTDP-D-glucose-4,6-dehydratase, RmlC: dTDP-4-dehydrorhamnose-3,5-epimerase, RmlD: dTDP-4-dehydrorhamnose-reductase, PCP: polysaccharide co-polymerase, Glc: glucose, GlcA: uronic acid, Man: mannose, Gal: galactose, Rha: rhamnose, UDP-glucose: UDP-Glc.
