# Supplementary material for: Identification of the Key Enzymes in WL Gum Biosynthesis and Critical Composition in Viscosity Control
Source: Front Bioeng Biotechnol. 2022 May 27;10:918687. doi: 10.3389/fbioe.2022.918687 (PMC9197254; doi:10.3389/fbioe.2022.918687)
Supplement: Supplementary file 1 [file DataSheet1.docx]

**Identification of the key enzymes in WL gum biosynthesis and crtitial composition for viscosity control**

Hui Li^1^, Jianlin Liu^1^, Zhongrui Guo^1^, Zaimei Zhang^1^, Mengqi Chen^1^, Benchao Li^1^, Han Xue^1^, Sixue Ji^1^, Hang Li ^1^, LingZhu^1^, Lijian Qin^1^, Jiqian Wang ^1^* and Hu Zhu ^1, 2, 3^*

**Supplementary Table 1 The strains and plasmids used in this work**

| Strains/ Plasmids | Genotype and/or relevant characteristics | Source |
| --- | --- | --- |
| *E. coli* DH5α | *supE*44 Δ*lac*U169(Φ80 *lacZ*ΔM15) *hsdR*17 *recA*1 *endA*1 *gyrA*96 *thi*-1 *relA*1 | Gibco-Brl |
| *Sphingomonas* sp. WG | Wild strain, Sm^R^ | This lab |
| pRK2013 | Km^R^, helper plasmid carrying RK2 *tra* genes, 48 kb | ([Ditta et al., 1980](#_ENREF_7)) |
| pBBR1MCS-3 | Tc^R^，LacZ-α mob rep | ([Kovach et al., 1995](#_ENREF_19)) |
| pBBR1MC3-*pgm* | Tc^R^, pBBR1MCS-3 derivative carrying the *pgm* gene | This lab |
| pBBR1MC3-*ugp* | Tc^R^, pBBR1MCS-3 derivative carrying the *ugp* gene | This lab |
| pBBR1MC3-*ugd* | Tc^R^, pBBR1MCS-3 derivative carrying the *ugd* gene | This lab |
| pBBR1MC3-*rmlA* | Tc^R^, pBBR1MCS-3 derivative carrying the *rmlA* gene | This lab |
| pBBR1MC3-*rmlB* | Tc^R^, pBBR1MCS-3 derivative carrying the *rmlB* gene | This lab |
| pBBR1MC3-*rmlC* | Tc^R^, pBBR1MCS-3 derivative carrying the *rmlC* gene | This lab |
| pBBR1MC3-*rmlD* | Tc^R^, pBBR1MCS-3 derivative carrying the *rmlD* gene | This lab |
| pBBR1MC3-*welB* | Tc^R^, pBBR1MCS-3 derivative carrying the *welB* gene | This lab |
| pBBR1MC3-*welK* | Tc^R^, pBBR1MCS-3 derivative carrying the *welK* gene | This lab |
| pBBR1MC3-*welL* | Tc^R^, pBBR1MCS-3 derivative carrying the *welL* gene | This lab |
| pBBR1MC3-*welQ* | Tc^R^, pBBR1MCS-3 derivative carrying the *welQ* gene | This lab |
| pBBR1MC3-*welS* | Tc^R^, pBBR1MCS-3 derivative carrying the *welS* gene | This lab |
| pBBR1MC3-*welC* | Tc^R^, pBBR1MCS-3 derivative carrying the *welC* gene | This lab |
| pBBR1MC3-*welE* | Tc^R^, pBBR1MCS-3 derivative carrying the *welE* gene | This lab |
| pBBR1MC3-*welR* | Tc^R^, pBBR1MCS-3 derivative carrying the *welR* gene | This lab |
| pBBR1MC3-*welI* | Tc^R^, pBBR1MCS-3 derivative carrying the *welI* gene | This lab |
| pBBR1MC3-*welJ* | Tc^R^, pBBR1MCS-3 derivative carrying the *welJ* gene | This lab |
| pBBR1MC3-*welM* | Tc^R^, pBBR1MCS-3 derivative carrying the *welM* gene | This lab |
| pBBR1MC3-*welN* | Tc^R^, pBBR1MCS-3 derivative carrying the *welN* gene | This lab |
| pBBR1MC3-*atrB* | Tc^R^, pBBR1MCS-3 derivative carrying the *atrB* gene | This lab |
| pBBR1MC3-*atrD* | Tc^R^, pBBR1MCS-3 derivative carrying the *atrD* gene | This lab |
| pBBR1MC3-*welF* | Tc^R^, pBBR1MCS-3 derivative carrying the *welF* gene | This lab |

**Reference:**

Ditta, G.; Stanfield, S.; Corbin, D.; Helinski, D. R., Broad host range DNA cloning system for gram-negative bacteria: construction of a gene bank of Rhizobium meliloti. *Proc Natl Acad Sci U S A* 1980, *77* (12), 7347-51.

Kovach, M. E.; Elzer, P. H.; Hill, D. S.; Robertson, G. T.; Farris, M. A.; Roop, R. M., 2nd; Peterson, K. M., Four new derivatives of the broad-host-range cloning vector pBBR1MCS, carrying different antibiotic-resistance cassettes. *Gene* **1995,** *166* (1), 175-6.

**Supplementary Table 2 Primers used for gene amplification**

| Gene | Gene length (bp) | Primers | Sequences | Restriction Enzymes |
| --- | --- | --- | --- | --- |
| *welL* | 867 | welLfor | TTAGGTACCGACCGCACCGCGCATCTCCGTTGTT | *Kpn* I |
|  |  | welLrev | TATGAGCTCCTACACCCTGAAGCCCAGCGACT | *Sac* I |
| *welB* | 1335 | welBfor | TTGGGTACCGATGTTGCGCAAGTCCGCGCTTCGGTTG | *Kpn* I |
|  |  | welBrev | GCTCTAGATCAGAAGGCGTTGGAGTGGACGATCACGCGCAG | *Xba* I |
| *ugp* | 870 | ugpfor | TTGGGTACCGATGACGATCAAGCCGCT | *Kpn* I |
|  |  | ugprev | GCTCTAGATCAACCGAGCGCACGCT | *Xba* I |
| *welQ* | 960 | welQfor | TATGGTACCGACCATCGTTATGACATCGCC | *Kpn* I |
|  |  | welQrev | TAATCTAGATCAGCCGCGGGGGCGGTCCAGAT | *Xba* I |
| *welC* | 1350 | welCfor | TATGGTACCGGTGAACATAGTCCAATTCTTTC | *Kpn* I |
|  |  | welCrev | GCTCTAGATTACTGTACTGCAACTCCCT | *Xba* I |
| *welJ* | 1386 | welJfor | TTACTCGAGGGTGGCTGTAGGTTCC | *Xho* I |
|  |  | welJrev | TATACTAGTTCAGCGGCTGGAAAGATCG | *Spe* I |
| *welS* | 1239 | welSfor | TATGGTACCGATGATCGTCGGATTCG | *Kpn* I |
|  |  | welSrev | TATGAGCTCTTAGATGATCGGTAT | *Sac* I |
| *welE* | 705 | welEfor | TTACTCGAGGATGGACGCGATCACTAC | *Xho* I |
|  |  | welErev | TATACTAGTTCAATATCCGTTCAGCAC | *Spe* I |
| *welR* | 2028 | welRfor | TATACTAGTGATGCTTACCATGCCGGAC | *Spe* I |
|  |  | welRrev | GCTCTAGATCAGACGTGGTGCAATTCC | *Xba* I |
| *welM* | 882 | welMfor | ATATATGGTACCGATGACCGCCACCGCGCGCACG | *Kpn* I |
|  |  | welMrev | TATTCTAGATCACGCCGGTTGAACATGCC | *Xba* I |
| *welN* | 690 | welNfor | TATACTAGTGATGATCGGCGCGGGCTGCTTC | *Spe* I |
|  |  | welNrev | TATTCTAGATCAGCTACGGGGCGGGTG | *Xba* I |
| *welI* | 906 | welIfor | TATCTCGAGGAAGAAGTTGTTTCTGGTGACG | *Xho* I |
|  |  | welIrev | TACGAGCTCTTACTGTGCTGAATTGCCGAC | *Sac* I |
| *welF* | 1194 | welFfor | TATGGTACCGATGTTTTTCGCGTCTGC | *Kpn* I |
|  |  | welFrev | TATTCTAGATCACAGCGATAGGGAAGTGGTCAG | *Xba* I |
| *rmlA* | 879 | rmlAfor | GCGGTACCGATGAAGGGTATCATTCTTG | *Kpn* I |
|  |  | rmlArev | TATACTAGTTCATGCTGCGGTCACC | *Spe* I |
| *rmlB* | 1062 | rmlBfor | TATGGTACCGATGCAGCAGACCTTCCT | *Kpn* I |
|  |  | rmlBrev | TAAGAGCTCTCAGGCGGTCTGGCCCAAC | *Sac* I |
| *rmlC* | 567 | rmlCfor | TAAGGTACCGACCCAAGTCCATCATCACG | *Kpn* I |
|  |  | rmlCrev | TATCTCGAGTCAGCCCTGATAGAAGAAG | *Xho* I |
| *rmlD* | 867 | rmlDfor | TTAACTAGTGATGCGTATCCTCGTC | *Spe* I |
|  |  | rmlDrev | TATTCTAGATCAGCGGGCGAGACGGTC | *Xba* I |
| *ugdG* | 1305 | Ugdgfor | CGGGGTACCGATTGGCACGGGCTATGT | *Kpn* I |
|  |  | Ugdgrev | CTCTAGATCAGTCGCGGCTCGGCTTGC | *Xba* I |
| *pgmG* | 1389 | Pgmgfor | CGGGGTACCGACGCATCGTTTCGACCCCACGTC | *Kpn* I |
|  |  | Pgmgrev | TATACTAGTTCAGTGGGCGGCCTGTTCG | *Spe* I |
| *atrB* | 2187 | atrBfor | TTAGGTACCGATGAAACGCGAAGAGATGC | *Kpn* I |
|  |  | atrBrev | TATACTAGTTCATGCCGCCATCTCCCCAAG | *Spe* I |
| *atrD* | 1395 | atrDfor | TATTCTAGAGATGAACGCGTTGGTC | *Xba* I |
|  |  | atrDrev | TATGAGCTCTTATCGTTCGTGCAGCGCGTCG | *Sac* I |


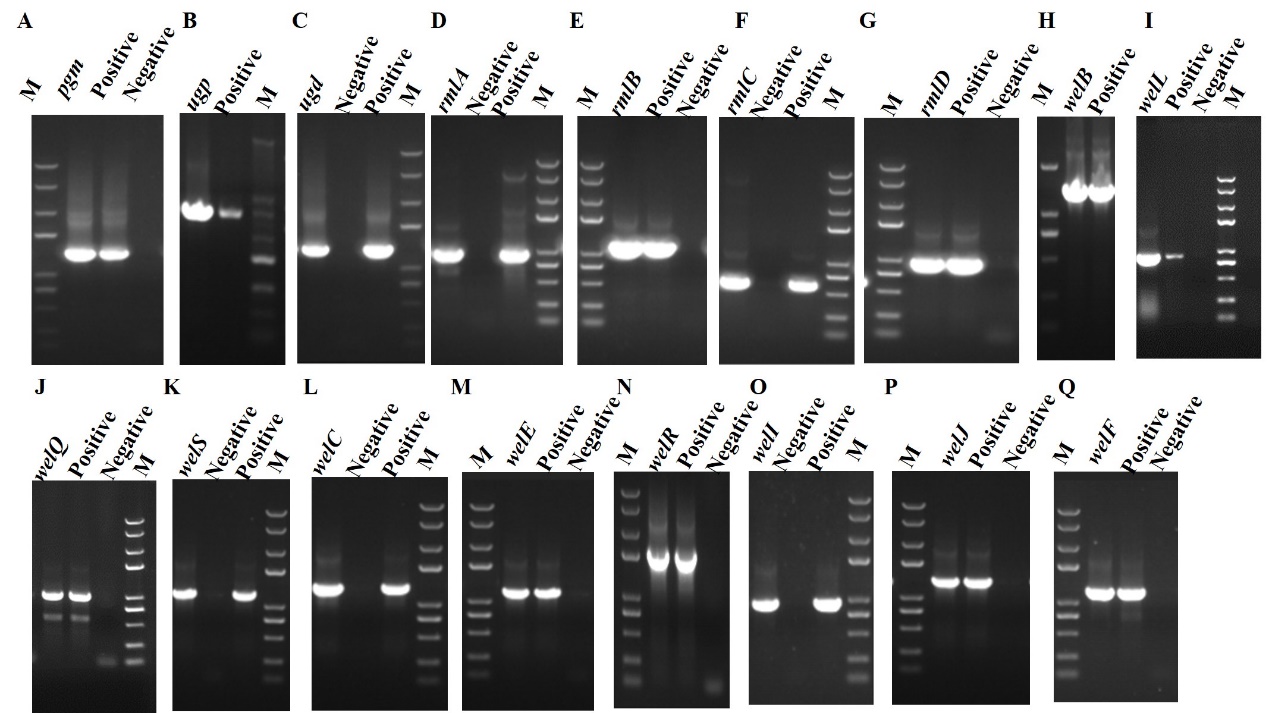


**Supplementary Figure 1 PCR identification of recombinant plasmids.** (A) *pgm*, (B) *ugp*, (C)*ugd*, (D) *rmlA*, (E) *rmlB*, (F) *rmlC*, (G) *rmlD*, (H) *welB*, (I) *well*, (J) *welQ*, (K) *welS*, (L) *welC*, (M) *welE*, (N) *welR*, (O) *welI*, (P) *welJ*,(Q) *welF*. M: DNA Marker D8000 with the bands at 8000 bp, 5000 bp, 3000 bp, 2000 bp, 1000 bp, 750 bp, 500 bp, 250 bp,125 bp. Positive control: *Sphingomonas* sp. WG as the template, negative control: water as the template


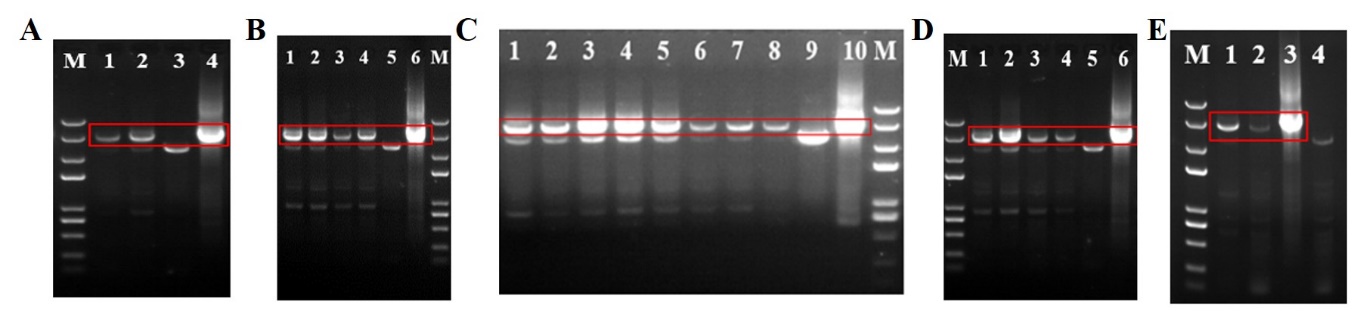


**Supplementary Figure 2 Verification of different over-expression strains.** (A) Identification of overexpression strains in group 1. M: DNA Marker D8000, Lane 1-2: *ugd, pgm over-expression strain*, 3: negative control using *Sphingomonas* sp. WG as the template, 4: positive control using pBBR1MCS-3 as the template. (B) Identification of over-expression strains in group 2. Lane 1-4: *rmlA* to *rmlD* over-expression strains, 5: negative control, 6: positive control. (C) Identification of over-expression strains in groups 3, 4, and 7. Lane 1-8: *welB*, *well, welQ, welS, welC, welE, welR, and welF over-expression strains*, 9: negative control, 10: positive control. (D) Identification of over-expression strains in group 5. Lane 1-4: *welI*, *welJ*, *welM*, *welN* over-expression strains, 5: negative control, 6: positive control. (E) Identification of over-expression strains in group 6. Lane 1-2: *atrB* and *atrD* overexpression strains, 3: negative control, 4: positive control


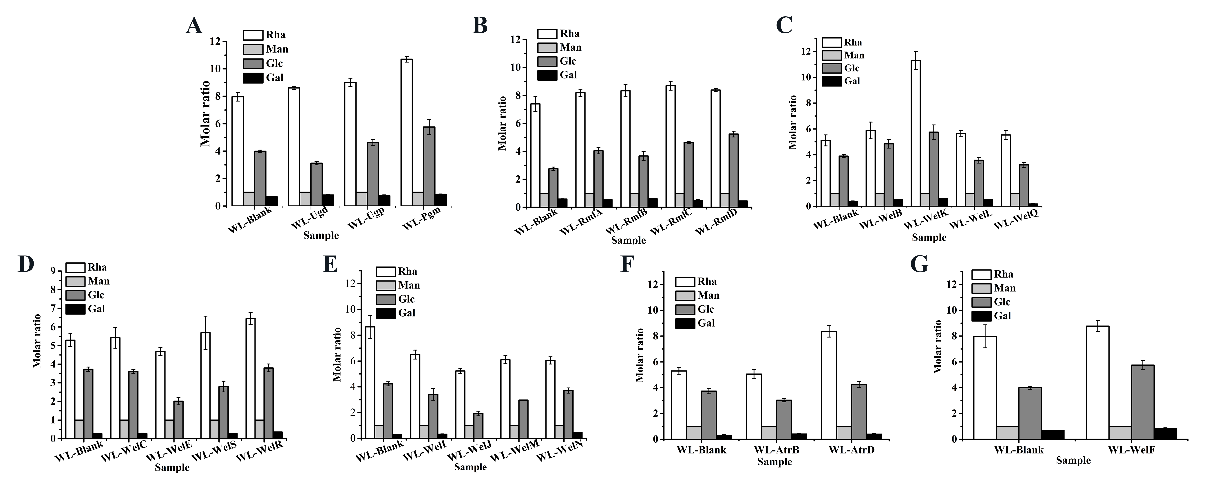


**Supplementary Figure 3 The effects of gene over-expression on neutral monosaccharide composition of EPS.** (A) The effects of genes in nucleotide-sugar UDP-glucose and UDP- glucuronic acid biosynthesis. (B) The effects of genes in nucleotide-sugar TDP-Rhamnose biosynthesis. (C) The effects of genes in repeating unit assembly. (D) The effects of genes in the polymerization process. (E) The effects of genes in exporting process. (F) The effects of ABC transporter protein AtrB and AtrD. (G) The effects of *welF*
